# Comparing the Antimicrobial Resistance Crisis to the COVID-19 Pandemic: A Randomized Public Health Messaging Experiment

**DOI:** 10.1093/cid/ciag110

**Published:** 2026-04-08

**Authors:** Alistair Thorpe, Rachael A Lee, Julia E Szymczak, Madeline C Farrell, Isabelle Palmer, William B Petty, Tyler Henderson, Angela Fagerlin, Valerie M Vaughn

**Affiliations:** Department of Population Health Sciences, Spencer Fox Eccles School of Medicine at University of Utah, Salt Lake City, Utah, USA; Department of Medicine, Division of Infectious Diseases, UAB School of Medicine, Birmingham, Alabama, USA; Department of Medicine, Division of Infectious Diseases, Birmingham VA Medical Center, Birmingham, Alabama, USA; Department of Internal Medicine, Division of Epidemiology, Spencer Fox Eccles School of Medicine at University of Utah, Salt Lake City, Utah, USA; Spencer Fox Eccles School of Medicine at University of Utah, Salt Lake City, Utah, USA; Department of Population Health Sciences, Spencer Fox Eccles School of Medicine at University of Utah, Salt Lake City, Utah, USA; Department of Psychology, Morehouse College, Atlanta, Georgia, USA; Department of Population Health Sciences, Spencer Fox Eccles School of Medicine at University of Utah, Salt Lake City, Utah, USA; Salt Lake City VA Informatics Decision-Enhancement and Analytic Sciences (IDEAS) Center for Innovation, Salt Lake City, Utah, USA; Department of Internal Medicine, Spencer Fox Eccles School of Medicine at University of Utah, Salt Lake City, Utah, USA

**Keywords:** health communication, antimicrobial resistance, public health, COVID-19, risk perceptions

## Abstract

**Background:**

Antimicrobial resistance (AMR) is an urgent health threat. Effective communication about AMR and avoiding antibiotics for viral infections is a public health priority. Here, we evaluated whether comparing the AMR crisis to the coronavirus disease 2019 (COVID-19) pandemic could enhance public understanding of AMR and reduce intentions to seek unnecessary antibiotics.

**Methods:**

In this randomized online survey (March–April 2024), US adults were randomized to receive 1 of 3 messages: (1) written message describing the AMR crisis (control); (2) written message comparing AMR to the COVID-19 pandemic; or (3) a series of poster-like graphics comparing AMR to the COVID-19 pandemic. Respondents then read a scenario describing a viral respiratory infection (where antibiotics are not clinically indicated) and asked to indicate whether they would (1) respond by visiting a primary care clinician and (2) desire to take antibiotics.

**Results:**

The final sample (N = 972, completion = 90%) had a mean age of 42 years and 58% identified as female. No statistically significant differences were found between messages for intention to visit a primary care clinician (*P* = .625) or desire to take antibiotics (*P* = .157). Across all respondents, older age and independent/third-party political affiliation were associated with lower antibiotic-seeking intentions, whereas medical maximizing, prior antibiotic use, being vaccinated for COVID-19, and pride in that status were associated with higher intentions.

**Conclusions:**

Comparing the threat of AMR to the COVID-19 pandemic did not reduce antibiotic-seeking intentions, indicating this analogy may not be an effective messaging strategy. Adults with greater care-seeking behaviors and preferences were more likely to report antibiotic-seeking intentions.

## Teaser

- Comparing the threat of antimicrobial resistance to the COVID-19 pandemic did not reduce antibiotic-seeking intentions
- Adults with stronger preferences for general and specific active health interventions were more likely to report antibiotic-seeking intentions

Antimicrobial resistance (AMR) is a major threat to global health [1], with >35 000 AMR-related deaths in the United States every year [2]. In response, public health organizations have launched numerous public-facing campaigns about AMR and the importance of avoiding unnecessary antibiotics [3–5] (eg, the US Centers for Disease Control and Prevention's “Be Antibiotics Aware” initiative, the US and World Antibiotic Awareness Weeks, the UK Health Security Agency's “Keep Antibiotics Working” campaign).

Unlike stewardship interventions that directly target prescribing [6], public-facing campaigns aim to address common concerns and misconceptions associated with antibiotic-seeking behaviors by informing people when antibiotics are necessary, the risks of unnecessary antibiotic use, and how to manage viral infections without antibiotics [3–5]. This upstream approach is important because antibiotic-seeking behaviors (eg, patients visiting primary care expecting to get antibiotics and making requests for them) can motivate clinicians to prescribe antibiotics even when they are not clinically necessary [7, 8].

Reducing unnecessary antibiotic use not only helps slow the spread of AMR but also can prevent patient harm from side effects and *Clostridioides difficile* [9, 10]. By providing accurate and actionable information, public health campaigns aim to empower people to manage mild viral symptoms at home to reduce their likelihood of antibiotic-associated harm. Scaled to population levels, a reduction in antibiotics for viral syndromes could also help ease pressure on healthcare systems and slow the spread of AMR.

Despite the ethical and clinical imperative to inform people about AMR, and the potential benefits of doing so, most campaigns have had limited impact on behavior and antibiotic prescription rates [11]. One often-cited reason for this is a lack of evidence-based strategies for communicating about AMR [4, 11]. AMR is a complex public health issue, and evidence on how to effectively share this information has lagged behind clinical advancements, resulting in persistent misunderstandings [12]. Even among members of the public who have heard of AMR, many see it as a distant threat (eg, for other countries, future generations) rather than a personal or immediate concern [5].

Although several studies have examined how clinically relevant information about antibiotics (eg, inefficacy for viral infections, possible side effects) can reduce unnecessary care-seeking and expectations [13–16], few studies have examined how to make the threat of AMR feel more personally relevant or meaningful [4]. To date, most studies seeking to communicate the risks of AMR have used fear-based messaging to highlight worst-case scenarios or emphasize personal risks [17, 18]. For example, a short film vividly depicting a dystopian future of untreatable infections due to AMR, told through a parent’s experience of personal loss and efforts to protect their child while in quarantine reduced viewers' intentions to expect and request antibiotics for a viral infection [18].

Analogies can make complex or unfamiliar health threats more understandable by relating them to familiar experiences or concepts [19–21]. The impacts of the coronavirus disease 2019 (COVID-19) pandemic prompted some researchers and public health experts to propose it as a reference for communicating the potential consequences of an AMR crisis [22]. Indeed, several online articles have used this comparison to emphasize the potential impact of AMR [23, 24]. Drawing this comparison may help people grasp the urgency and scale of AMR and motivate them to take action. However, although analogies have been successful health communication strategies in other health contexts (eg, vaccination) [19], it is critical to test the efficacy of such comparisons before implementing them in new settings to prevent unanticipated outcomes (eg, underestimating the threat of the condition, associating it with inappropriate treatments or behaviors) [25]. Thus, we created and tested public health messages comparing the AMR crisis to the COVID-19 pandemic. We hypothesized that relative to a control message, comparing the AMR crisis to the COVID-19 pandemic would decrease reported (1) intention to visit a primary care clinician and (2) desire to take antibiotics for a viral respiratory infection (Hypothesis 1). As graphics have been shown to aid health-related understanding and motivate behavioral intentions [26], we also hypothesized that visual messages in the form of poster-like graphics would be more effective than text-based messages at decreasing care-seeking intentions and antibiotic desires for a viral respiratory infection (Hypothesis 2). Last, we hypothesized that relative to a control message, comparing the AMR crisis to the COVID-19 pandemic would not affect reported intention to take antibiotics for a bacterial infection.

## METHODS

### Study Population and Recruitment

This study was deemed exempt by the Institutional Review Board at the University of Utah (IRB_00167676). Online survey respondents were recruited from 21 March through 23 April 2024 by Dynata, a commercial market research company with diverse pools of individuals who have agreed to be invited to take part in online survey studies. Dynata sent email invites to eligible individuals with a link directing them to an information page, which informed respondents of their choice to voluntarily opt in or out, with consent implied by continuing the survey. Respondents were compensated based on the terms of their agreement with Dynata.

Eligibility: the survey was open to US adults aged ≥18 years who could speak English and access the online survey. To ensure enough power to detect a small effect of the messages (*f* = .10, with 1-β = .95, and α = .05 for a 1-way analysis of variance), we aimed to recruit 1000 respondents (∼330/study arm) with quotas for age in years (18–33, 34–49, 50–64 [33% each]), gender identity (female [49%], male [49%], any other gender identity [1%]), racial/ethnic identity (non-Hispanic White [30%], non-Hispanic Black [30%], Hispanic [30%], any other race or ethnicity [10%]), and US Census Region (Northeast [17%], Midwest [21%], South [39%], West [24%]).

### Procedure

**Figure 1.**
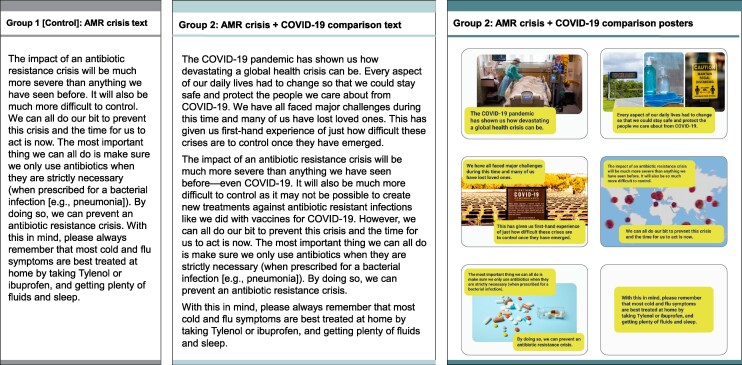
Experimental study arm assignment.

In this between-groups experiment, respondents were randomized to either a control group or 1 of 2 intervention groups (see Figure 1 and the supplement for a full description of the survey items including the messages shown to each group). Prior to seeing the messages, respondents all saw the same introductory text, “There is a global health threat posed by bacteria that cannot be treated with antibiotics (ie, antibiotic resistance). Please carefully consider the following information about antibiotics and antibiotic resistance.”

**Group 1 [Control]:**  *AMR crisis text*. Respondents saw a written message about an AMR crisis (see Figure 1). The message was designed by the study team to reflect public health messages about the global health threat posed by AMR and what the public can do to help.

**Group 2:**  *AMR crisis + COVID-19 comparison text*. Respondents saw the control message with additional text drawing comparisons with the COVID-19 pandemic.

**Group 3:**  *AMR crisis + COVID-19 comparison posters.* Respondents saw the same information as group 2, but on a series of posters with accompanying images.

After receiving their message, all respondents were shown a scenario describing symptoms of a viral respiratory infection where antibiotics are not clinically indicated [27]. They were then asked: “At this point, do you think you would go to see a primary care clinician about these symptoms?” (ie, intention to seek care) and “Would you want to get antibiotics for these symptoms?” (ie, desire for antibiotics) on a 4-point scale (1 = “Definitely WOULD NOT”, 2 = “Probably WOULD NOT”, 3 = “Probably WOULD”, 4 = “Definitely WOULD”).

To assess whether the messages might unintentionally dissuade people from taking clinically necessary antibiotics, respondents were also shown a second scenario describing symptoms of a bacterial kidney infection where a clinician prescribed them a 7-day course of antibiotics and asked whether they would take the antibiotics (using the same 4-point scale).

After the second scenario, respondents saw additional questions regarding COVID-19 and AMR, then self-reported individual psychological characteristics (eg, medical maximizing) and demographic characteristics.

### Preregistered Analyses

To test our hypotheses, we ran three 1-way analyses of variance to examine the impact of the messages on care-seeking intentions, antibiotic desires, and adherence. We also assessed whether age or gender identity would moderate any effect of the interventions because prior research shows that antibiotic-related intentions and patterns of use often vary across these groups [28]. Analyses were performed using RStudio 4.2.2 with statistical significance set at. 05 (2-sided).

### Exploratory Analyses

As a sensitivity check, we repeated the primary analyses using nonparametric alternatives. We also examined whether any impact of the messages might depend on the how long respondents spent viewing them. To explore this possibility, we reran our preregistered analyses on 2 subsets: respondents who spent ≥10 seconds on the single page with the text messages (groups 1 and 2) and those who spent ≥30 seconds on the 3-page series of posters (group 3). These are thresholds used in similar public health messaging studies [26] and correspond to approximately one-third of the average viewing time.

Given evidence that recollections of the COVID-19 pandemic (eg, perceived infection risk) differ by vaccination status, identification with that status, and political affiliation [29] we reran our preregistered analyses with 3 moderators: COVID-19 vaccination status (vaccinated vs not), pride in that vaccination status, and political party affiliation (Democrat, Republican, Independent/third party, none disclosed).

Last, given the null effect of the messages, we ran 2 multiple regressions to identify factors associated with care-seeking intentions and antibiotic desires in the viral respiratory infection scenario using the full sample. Both models used the same predictors, which sought to capture a range of demographic characteristics, health status and behavior measures, and individual differences. The demographic characteristics included age (18–33, 34–49, 50–64 years), gender identity (female, male), racial/ethnic identity (non-Hispanic White, non-Hispanic Black, Hispanic, Any other), US census region (Northeast, Midwest, South, West), residence (rural, suburban, urban), and educational attainment (≤high school, Bachelor's degree, ≥Bachelor's degree). Health status and behaviors included antibiotic use over the past 12 months (0, 1, 2, 3, 4, ≥5 times), number of comorbid conditions (0–12), and COVID-19 vaccination status (vaccinated vs not). Individual difference characteristics included health literacy needs, subjective numeracy, political stance on social issues, political party affiliation, worry about short-time antibiotic side effects, worry about long-time antibiotic side effects, pride in COVID-19 vaccination status, medical maximizing, and disbelief in science.

## RESULTS

Of the 1136 eligible respondents who started the survey, 1035 (91%) completed the survey. We then excluded 62 respondents whose free-text answers led us to question the validity of their data and 1 who finished in ≤5 minutes, resulting in a final sample of 972. The final sample had a mean age of 42 years and 58% identified as female (Table 1). Survey completion times [Mins:Secs] did not differ by study arm (16:46 vs 16:7 vs 17:37, *P* = .964) with a median time of 16 minutes overall.

**Table 1.** Self-reported Respondent Demographic Characteristics, Health Status, Health Behaviors, and Individual Characteristics

Characteristic	Overall (N = 972)	Group 1 (n = 315)	Group 2 (n = 332)	Group 3 (n = 325)
Mean age ± standard deviation – years	42 (13)	42 (13)	41 (13)	42 (13)
Age category – no. (%)	…	…	…	…
18–33	316 (32.5)	99 (31.4)	114 (34.3)	103 (31.7)
34–49	321 (33.0)	108 (34.3)	106 (31.9)	107 (32.9)
50–64	335 (34.5)	108 (34.3)	112 (33.7)	115 (35.4)
Gender identity – no. (%)	…	…	…	…
Female	560 (57.6)	182 (57.8)	178 (53.6)	200 (61.5)
Male	399 (41.0)	130 (41.3)	147 (44.3)	122 (37.5)
Any other identity	13 (1.3)	3 (1.0)	7 (2.1)	3 (0.9)
Racial/ethnic identity – no. (%)	…	…	…	…
Hispanic	340 (35.0)	112 (35.6)	112 (33.8)	116 (35.7)
Non-Hispanic Black	352 (36.3)	109 (34.6)	121 (36.6)	122 (37.5)
Non-Hispanic White	176 (18.1)	55 (17.5)	70 (21.1)	51 (15.7)
Any other identity	103 (10.6)	39 (12.4)	28 (8.5)	36 (11.1)
US Census Region – no. (%)	…	…	…	…
Northeast	166 (17.1)	43 (13.7)	67 (20.2)	56 (17.4)
Midwest	160 (16.5)	57 (18.2)	46 (13.9)	57 (17.7)
South	441 (45.6)	142 (45.2)	157 (47.3)	142 (44.1)
West	201 (20.8)	72 (22.9)	62 (18.7)	67 (20.8)
Residence	…	…	…	…
Rural	238 (24.5)	76 (24.2)	95 (28.6)	67 (20.7)
Suburban	421 (43.4)	137 (43.6)	135 (40.7)	149 (46.0)
Urban	311 (32.1)	101 (32.2)	102 (30.7)	108 (33.3)
Educational attainment	…	…	…	…
High school or less	244 (25.2)	89 (28.3)	82 (24.8)	73 (22.5)
Some college or trade	316 (32.6)	102 (32.5)	103 (31.1)	111 (34.2)
≥Bachelor's	410 (42.3)	123 (39.2)	146 (44.1)	141 (43.4)

### Scenario: Hypothetical Viral Respiratory Infection Symptoms

**Figure 2.**
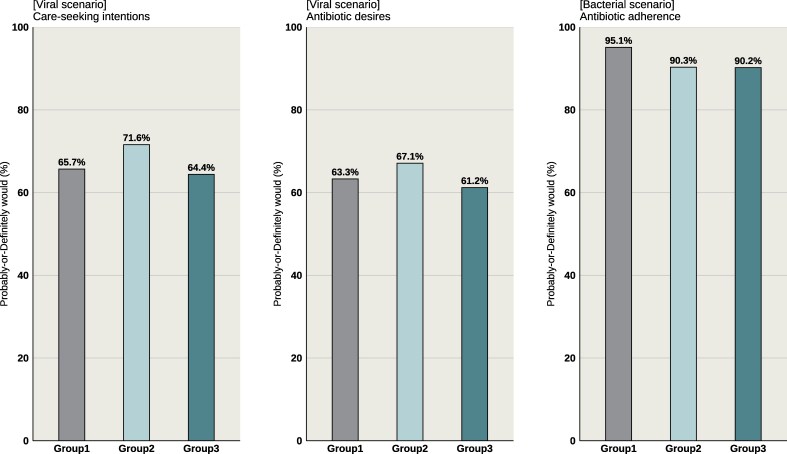
Primary outcome measures by study arm. Group 1 = AMR crisis text, Group 2 = AMR crisis text + COVID-19 comparison text, Group 3 = AMR crisis + COVID-19 comparison posters. Abbreviations: AMR, antimicrobial resistance; COVID-19, coronavirus disease 2019.

Overall, 67% of respondents indicated that they probably or definitely would visit a primary care clinician (group 1 = 65.7% vs group 2 = 71.6% vs group 3 = 64.4%; *P* = .625) and 64% indicated that they probably-or-definitely would want to take antibiotics (63.3% vs 67.1% vs 61.2%; *P* = .157) based on the symptoms described in the scenario (Figure 2), with no statistically significant differences between experimental groups (Table 2).

**Table 2.** Outcome Measures Overall and According to Group Assignment

	Values for Final Sample						Values for Subgroup Who Spent ≥10 (or ≥30) Seconds With the Messages on Their Screen					
Care-seeking intentions [V]*	Overall (N = 972)	Group 1 (n = 315)	Group 2 (n = 332)	Group 3 (n = 325)	Test Statistic	*P* Value	Overall (n = 488)	Group 1 (n = 147)	Group 2 (n = 141)	Group 3 (n = 200)	Test Statistic	*P* Value
Mean ± SD	2.88 ± 0.99	2.89 ± 1.00	2.91 ± 0.95	2.84 ± 1.04	F(2966) = 0.47	.625^a^	2.57 ± 1.04	2.52 ± 1.06	2.72 ± 1.00	2.52 ± 1.06	F(2485) = 2.75	.065^a^
Median (IQR)	3 (2,4)	3 (2,4)	3 (2,4)	3 (2,4)	χ^2^ (2) = 0.56	.755^b^	3 (2,3)	3 (2,3)	3 (2,3)	3 (2,3)	χ^2^ (2) = 4.01	.135^b^
**Antibiotic** **desires [V]** *							**…**					
Mean ± SD	2.77 ± 1.01	2.79 ± 1.02	2.83 ± 0.99	2.68 ± 1.02	F(2969) = 1.86	.157^a^	2.81 ± 1.02	2.71 ± 1.08	2.98 ± 0.87	2.77 ± 1.06	F(2485) = 1.86	.157^a^
Median (IQR)	3 (2,4)	3 (2,4)	3 (2,4)	3 (2,4)	χ^2^ (2) = 3.55	.170^b^	3 (2,4)	3 (2,4)	3 (2,4)	3 (2,4)	χ^2^ (2) = 3.54	.171^b^
**Antibiotic** **adherence [B]** *							**…**					
Mean ± SD	3.56 ± 0.72	3.63 ± 0.64	3.53 ± 0.73	3.53 ± 0.78	F(2969) = 2.19	.112^a^	3.71 ± 0.62	3.74 ± 0.57	3.74 ± 0.51	3.67 ± 0.72	F(2485) = 0.82	.442^a^
Median (IQR)	4 (3,4)	4 (3,4)	4 (3,4)	4 (3,4)	χ^2^ (2) = 2.88	.237^b^	4 (4,4)	4 (2,4)	4 (2,4)	4 (2,4)	χ^2^ (2) = 0.24	.888^b^

IQR, interquartile range; SD, standard deviation.

^a^Ominbus 1-way analysis of variance was used for this comparison (parametric test).

^b^Kruskal-Wallis rank sum was used for this comparison (nonparametric test), [V] = Viral scenario, [B] = Bacterial scenario.

^*^All outcomes measured on a 4-point Likert scale.

### Scenario: Antibiotics Prescribed for a Bacterial Kidney Infection

Most respondents (92%) indicated they would take the antibiotics prescribed by the clinician. We found no statistically significant differences between groups (95.1% vs 90.3% vs 90.2%; *P* = .112). All preregistered findings were robust to nonparametric tests and when controlling for respondents’ age or gender identity (Tables 2 and 3).

**Table 3.** Outcome Measures According to Group Assignment and Controlling for Age, Gender Identity, and COVID-19 Vaccination Status and Identification

…	Viral Scenarios				Bacterial Scenario	
	Care-seeking Intentions		Antibiotic Desires		Antibiotic Adherence	
	Test Statistic	*P* Value	Test Statistic	*P* Value	Test Statistic	*P* Value
**Model 1: Age**	…		…	…	…	…
Group	F(2) = 0.40	.671	F(2) = 1.65	.192	F(2) = 2.17	.115
Age	F(1) = 9.56	.**002**	F(1) = 27.41	**<**.**001**	F(1) = 1.32	.251
Group*Age	F(2) = 0.62	.540	F(2) = 0.24	.787	F(2) = 0.15	.859
**Model 2: Gender identity**	…		…	…	…	…
Group	F(2) = 0.19	.829	F(2) = 1.54	.214	F(2) = 2.19	.112
Gender identity	F(1) = 0.07	.784	F(1) = 0.28	.594	F(1) = 0.81	.370
Group*Gender identity	F(2) = 0.63	.534	F(2) = 0.44	.641	F(2) = 1.06	.347
**Model 2: Political party affiliation**	…		…	…	…	…
Group	F(2) = 1.51	.221	F(2) = 3.77	.**023**	F(2) = 1.02	.362
Political party affiliation	F(3) = 1.82	.143	F(3) = 4.37	.**005**	F(3) = 2.67	.**047**
Group*Political party affiliation	F(6) = 1.06	.387	F(6) = 0.99	.428	F(6) = 0.47	.829
**Model 3: COVID-19 vaccination status and pride**		…		…	…	…
Group	F(2) = 0.98	.376	F(2) = 1.04	.353	F(2) = 0.16	.852
Vaccination status	F(1) = 0.01	.940	F(1) = 0.02	.880	F(1) = 0.10	.746
Pride in vaccination status	F(1) = 0.98	.322	F(1) = 0.43	.510	F(1) = 0.06	.810
Group*Vaccination status	F(2) = 0.89	.413	F(2) = 0.71	.490	F(2) = 0.82	.442
Group*Pride in vaccination status	F(2) = 1.38	.252	F(2) = 0.65	.524	F(2) = 0.08	.922
Group*Vaccination status*Pride	F(1) = 1.21	.271	F(1) = 1.19	.275	F(1) = 0.84	.359

^*^Interaction effect, otherwise row results report main effects, *P* values < .05 are shown in bolded font.

### Exploratory Results

Results from the subgroup of respondents (n = 488) who viewed the messages for at least one-third of the average viewing time did not differ from the main analysis (care-seeking intentions *P* = .065, desires to take antibiotic *P* = .157, or adherence *P* = .442). We found no significant interactions between the impact of the messages and respondents’ COVID-19 vaccination status, pride in that status, and political party affiliation (Tables 2 and 3, and Supplementary Figures 1–4).

**Figure 3.**
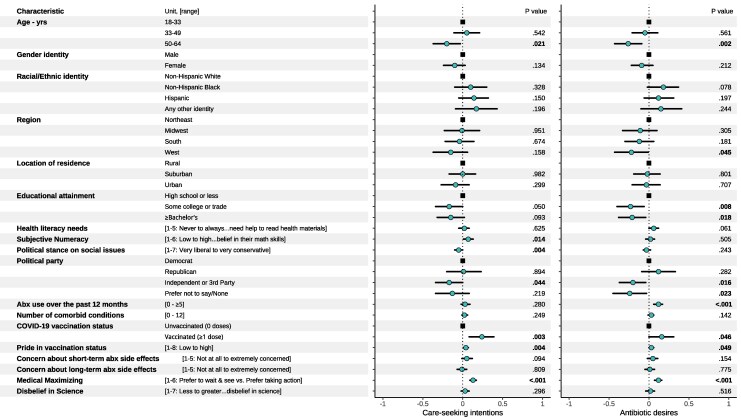
Factors associated with care-seeking intentions and antibiotic desires for a viral respiratory infection, multiple regression.

Respondents aged 50–64 (vs 18–33) years and Independent/third-party affiliation (vs Democrat) predicted both a lower likelihood of care-seeking intentions and antibiotic desires (Figure 3). In contrast, medical maximizing (preferring to act in ambiguous clinical situations) and being vaccinated for COVID-19 (vs not) and pride in that vaccination status was associated with greater care-seeking intentions and desires for antibiotics.

Unique predictors of care-seeking intentions included higher subjective numeracy and more liberal outlook on social issues. For desires to take antibiotics, US census region (West vs Northeast), educational attainment (more than a college or trade vs high school or less), and political affiliation (none disclosed vs Democrat) predicted lower desires, whereas more frequent antibiotic use over the past 12 months predicted greater desires (Supplementary Table 1).

## DISCUSSION

AMR is a critical global health threat fueled in part by clinically unnecessary antibiotic use. Effective communication strategies are essential to support large-scale public health efforts. In this randomized online experiment, we tested whether comparing AMRs potential consequences with an experienced health crisis—an evidence-based strategy assessed in other health contexts [19–21]—could promote judicious antibiotic-seeking behaviors. Contrary to expectations, the messages did not reduce intentions to visit a primary care clinician or desires to take antibiotics for viral respiratory symptoms. These findings suggest that drawing parallels between AMR and COVID-19 may not represent an effective public health messaging strategy.

Like others who have advocated for and used this comparison [22–24], we expected that framing the threat of AMR through an experienced health crisis might help people relate to and internalize its impact. However, the null findings remained consistent even after controlling for age, gender identity, political party affiliation, COVID-19 vaccination status and pride in that status, and time spent viewing the messages. Given our hope that this comparison could be a low-cost way to strengthen public-facing campaigns, these findings are disappointing. However, it is a valuable insight that this comparison may not be worth employing in future campaigns, allowing designers to focus time and resources on more promising strategies (eg, reducing diagnostic uncertainty [13, 16], leveraging trust in providers [15, 30], emphasizing the consequences and personal impacts of AMR [16, 17]).

One explanation for our negative findings is that too much time has passed since the COVID-19 pandemic, particularly as concerns of disengagement with public health recommendations due to prolonged stress and restrictions (ie, pandemic fatigue) arose within the first year of the pandemic [31]. By the time of our study, respondents may have forgotten the severity of their experiences or simply no longer cared or had strong negative reactions to the COVID-19 response. Despite the efforts of public health experts to stress the likelihood of future health crises, some respondents may have doubted the AMR comparison. Reports indicate a substantial number of people see COVID-19 as a unique, 1-off event, and do not worry about another crisis within their lifetime [32]. As many people held strong beliefs about the origin and threat posed by COVID-19, some respondents may have felt the risks and impact of the COVID-19 pandemic were exaggerated by the media and public health institutions [33], which could have led them to then underestimate the risks of AMR when the 2 were compared. It is also possible that elements of alarmist framing across all conditions, including the control, may have reduced the potential for differential effects. Although the messages were unable to promote judicious antibiotic-seeking behaviors, it was reassuring that we observed no significant negative impacts of the messages on clinically necessary antibiotic use. In the scenario where the clinician prescribed antibiotics for a bacterial infection, adherence remained high across groups (≥90%).

The regression analyses revealed patterns of predictors worth noting. Respondents who reported stronger tendencies toward active health intervention (eg, those high on medical maximizing, vaccinated for COVID-19 and proud of that status, more frequent prior antibiotic use) were more likely to want to visit a primary care clinician and take antibiotics when not clinically necessary (ie, for a viral respiratory infection). These same factors often predicted engagement in protective and pro-social health behaviors during the pandemic (eg, masking, vaccine uptake) [34, 35]. However, in the context of antibiotic use, they appear to be associated with behaviors that increase the risk of unnecessary antibiotic exposure and its harms. Given the overlap in predictors of care-seeking and antibiotic desires, our findings reinforce the need for upstream strategies to reduce unnecessary care-seeking. An important implication is that these more healthcare-oriented individuals may be more receptive to educational efforts and alternative strategies (eg, delayed prescribing [36]), creating a valuable opportunity to explore how best to engage with this population.

Respondents aged ≥50 years were consistently less likely to report intentions to visit a primary care clinician and to want antibiotics when not clinically necessary. These findings align with reports of greater caution about antibiotic use in adults aged ≥50 years [37]. Prior qualitative research suggests that although older adults may be adept at interpreting their symptoms accurately, they may also be prone to minimize their symptoms and delay care unless symptoms become intolerable or family members encourage them to [38].

This study has several limitations. Although self-reports are generally reliable predictors of health behaviors [39], respondents may not have given fully accurate answers (eg, due to social desirability bias). Elements of the study's design (eg, nonrepresentative, US-based, online, and English only) restrict generalization to other settings and populations including those without internet access or with lower English proficiency. Additionally, the hypothetical nature of the messaging and scenarios meant respondents were not experiencing symptoms or engaging in a real-life clinical interaction and the absence of pretesting limits our understanding as to how respondents interpreted the materials. However, this vignette-based approach has been highly valuable for simulating realistic healthcare scenarios, whereas allowing the systematic manipulation of specific factors under controlled conditions without the ethical and practical challenges of altering actual clinical encounters [27]. As healthcare increasingly shifts toward virtual and asynchronous platforms such as telehealth and patient portal messaging [40], where patients make decisions without in-person interaction, these vignette-based designs remain highly relevant for understanding patient behavior. Future studies using vignette-based methods to explore how differences in messaging and clinical context shape antibiotic care-seeking intentions could therefore provide actionable guidance for both clinician–patient discussions and public health messaging.

### Conclusion

Comparing the threat of AMR to the experienced impacts of the COVID-19 pandemic does not appear to be an effective communication strategy. Respondents with greater care-seeking behaviors and preferences reported being more likely to visit a primary care clinician and want antibiotics even when they were not clinically necessary. These findings can help inform communication strategies seeking to promote judicious antibiotic use.

## Supplementary Material

ciag110_Supplementary_Data

## Supplementary Data

Supplementary materials are available at *Clinical Infectious Diseases* online. Consisting of data provided by the authors to benefit the reader, the posted materials are not copyedited and are the sole responsibility of the authors, so questions or comments should be addressed to the corresponding author.

## References

[1] Larkin H . Increasing antimicrobial resistance poses global threat, WHO says. JAMA 2023; 329:200. 10.1001/jama.2022.23552 36648459

[2] 2019 Antibiotic Resistance Threats Report. Antimicrobial Resistance. CDC. Available at: https://www-cdc-gov.ezproxy.lib.utah.edu/antimicrobial-resistance/data-research/threats/index.html. Accessed 6 June 2025.

[3] Huttner B, Goossens H, Verheij T, Harbarth S. Characteristics and outcomes of public campaigns aimed at improving the use of antibiotics in outpatients in high-income countries. Lancet Infect Dis 2010; 10:17–31. 20129146 10.1016/S1473-3099(09)70305-6

[4] Huttner B, Saam M, Moja L, et al How to improve antibiotic awareness campaigns: findings of a WHO global survey. BMJ Glob Health 2019; 4:e001239. 10.1136/bmjgh-2018-001239 PMC6528771 31179029

[5] McCullough AR, Parekh S, Rathbone J, Del Mar CB, Hoffmann TC. A systematic review of the public's knowledge and beliefs about antibiotic resistance. J Antimicrob Chemother 2016; 71:27–33. 26459555 10.1093/jac/dkv310

[6] Cosgrove SE, Srinivasan A. Antibiotic stewardship: a decade of progress. Infect Dis Clin 2023; 37:659–67. 10.1016/j.idc.2023.06.003 PMC11320720 37537002

[7] McNulty CA, Nichols T, French DP, Joshi P, Butler CC. Expectations for consultations and antibiotics for respiratory tract infection in primary care: the RTI clinical iceberg. Br J Gen Pract 2013; 63:e429–36. 23834879 10.3399/bjgp13X669149 PMC3693799

[8] Sirota M, Round T, Samaranayaka S, Kostopoulou O. Expectations for antibiotics increase their prescribing: causal evidence about localized impact. Health Psychol 2017; 36:402–9. 28206788 10.1037/hea0000456

[9] Stevens V, Dumyati G, Fine LS, Fisher SG, van Wijngaarden E. Cumulative antibiotic exposures over time and the risk of Clostridium difficile infection. Clin Infect Dis 2011; 53:42–8. 21653301 10.1093/cid/cir301

[10] Curran J, Lo J, Leung V, et al Estimating daily antibiotic harms: an umbrella review with individual study meta-analysis. Clin Microbiol Infect 2022; 28:479–90. 34775072 10.1016/j.cmi.2021.10.022

[11] Gilham EL, Pearce-Smith N, Carter V, Ashiru-Oredope D. Assessment of global antimicrobial resistance campaigns conducted to improve public awareness and antimicrobial use behaviours: a rapid systematic review. BMC Public Health 2024; 24:396. 38321479 10.1186/s12889-024-17766-w PMC10848528

[12] Krockow EM, Jenkins DR, Mkumbuzi S, Flusberg SJ, Tarrant C. Why antimicrobial resistance messaging fails: qualitative insights interpreted through the elaboration likelihood model. JAC-Antimicrob Resist 2025; 7:dlaf148. 40799680 10.1093/jacamr/dlaf148 PMC12342773

[13] Thorpe A, Sirota M, Juanchich M, Orbell S. Action bias in the public's clinically inappropriate expectations for antibiotics. J Exp Psychol Appl 2020; 26:422–31. 32271052 10.1037/xap0000269

[14] Theodoropoulou A, Lisi M, Rolision J, Sirota M. The effects of communicating illness diagnostic and treatment information and C-reactive protein test results on people's antibiotic expectations. Br J Health Psychol 2025; 30:e70020. 40891384 10.1111/bjhp.70020 PMC12403044

[15] Thorpe A, Sirota M, Juanchich M, Orbell S. Always take your doctor's advice’: does trust moderate the effect of information on inappropriate antibiotic prescribing expectations? Br J Health Psychol 2020; 25:358–76. 32196870 10.1111/bjhp.12411

[16] Sievert EDC, Korn L, Gross M, Santana AP, Böhm R, Betsch C. Communicating diagnostic uncertainty reduces expectations of receiving antibiotics: two online experiments with hypothetical patients. Appl Psychol Health Well-Being 2024; 16:1459–78. 38500005 10.1111/aphw.12536

[17] Roope LSJ, Tonkin-Crine S, Herd N, et al Reducing expectations for antibiotics in primary care: a randomised experiment to test the response to fear-based messages about antimicrobial resistance. BMC Med 2020; 18:110. 32321478 10.1186/s12916-020-01553-6 PMC7178623

[18] Sirota M, Juanchich M. Seeing an apocalyptic post-antibiotic future lowers antibiotics expectations and requests. Commun Med 2024; 4:141. 38997505 10.1038/s43856-024-00567-y PMC11245540

[19] Scherer AM, Scherer LD, Fagerlin A. Getting ahead of illness: using metaphors to influence medical decision making. Med Decis Making 2015; 35:37–45. 24615273 10.1177/0272989X14522547

[20] Edwards A . Communicating risks through analogies. BMJ 2003; 327:749. 14512490 10.1136/bmj.327.7417.749 PMC200820

[21] Galesic M, Garcia-Retamero R. Using analogies to communicate information about health risks. Appl Cogn Psychol 2013; 27:33–42.

[22] Murray AK . The novel coronavirus COVID-19 outbreak: global implications for antimicrobial resistance. Front Microbiol 2020; 11:1020. 32574253 10.3389/fmicb.2020.01020 PMC7237633

[23] Benson T . There's another pandemic-level health threat slowly building—this one from bacteria. Business Insider.Available at: https://www.businessinsider.com/bacteria-pandemic-threat-after-covid-coronavirus-2020-10. Accessed 9 October 2025.

[24] Antimicrobial resistance will be worse than COVID—we have to act now. Available at: https://thehill.com/opinion/healthcare/4052215-antimicrobial-resistance-will-be-worse-than-covid-we-have-to-act-now/. Accessed 9 October 2025.

[25] Bruine de Bruin W, Carman KG, Parker AM. Mental associations with COVID-19 and how they relate with self-reported protective behaviors: a national survey in the United States. Soc Sci Med 1982 2021; 275:113825. 10.1016/j.socscimed.2021.113825 PMC7937328 33735777

[26] Thorpe A, Fagerlin A, Butler J, et al Communicating about COVID-19 vaccine development and safety. PLoS One 2022; 17:e0272426. 35930557 10.1371/journal.pone.0272426 PMC9355181

[27] Hillen MA, Visser LNC, Labrie N, et al Development of GROVE: a guideline for Reporting vignette experiments conducted in a healthcare context. Patient Educ Couns 2025; 136:108750. 40107182 10.1016/j.pec.2025.108750 PMC12961645

[28] Thorpe A, Lee RA, Fagerlin A, Vaughn VM, Szymczak JE. Characteristics and antibiotic preferences of US adults reporting frequent use vs no use of antibiotics. JAMA Netw Open 2025; 8:e251429. 40116831 10.1001/jamanetworkopen.2025.1429 PMC11929022

[29] Sprengholz P, Henkel L, Böhm R, Betsch C. Historical narratives about the COVID-19 pandemic are motivationally biased. Nature 2023; 623:588–93. 37914928 10.1038/s41586-023-06674-5

[30] Gilham EL, Casale E, Hardy A, et al Assessing the impact of a national social marketing campaign for antimicrobial resistance on public awareness, attitudes, and behaviour, and as a supportive tool for healthcare professionals, England, 2017 to 2019. Eurosurveillance 2023; 28:2300100. 37997667 10.2807/1560-7917.ES.2023.28.47.2300100 PMC10668255

[31] Pandemic fatigue—reinvigorating the public to prevent COVID-19: policy framework for supporting pandemic prevention and management. Available at: https://iris.who.int/handle/10665/335820. Accessed 9 October 2025.

[32] Gallup Inc . Pandemic deemed “over” by 59%, yet future health crisis feared. Gallup.com. March 2025. Available at: https://news.gallup.com/poll/657821/pandemic-deemed-yet- future-health-crisis-feared.aspx. Accessed 10 October 2025.

[33] Tyson A, Lipka M, Deane C. 5 Years later: America looks back at the impact of COVID-19. Pew Research Center. 12 February 2025. Available at: https://www.pewresearch.org/politics/2025/02/12/5-years-later-america-looks-back-at-the-impact-of-covid-19/. Accessed 10 October 2025.

[34] Thorpe A, Zhong L, Scherer LD, Drews FA, Shoemaker H, Fagerlin A. Demographic, structural, and psychological predictors of risk-increasing and mask wearing behaviors among US adults between December 2020–March 2021. Patient Educ Couns 2023; 114:107792. 37201301 10.1016/j.pec.2023.107792

[35] Thorpe A, Fagerlin A, Drews FA, Shoemaker H, Brecha FS, Scherer LD. Predictors of COVID-19 vaccine uptake: an online three-wave survey study of US adults. BMC Infect Dis 2024; 24:304. 38475702 10.1186/s12879-024-09148-9 PMC10936026

[36] Spurling GK, Del Mar CB, Dooley L, Foxlee R, Farley R. Delayed antibiotic prescriptions for respiratory infections. Cochrane Acute Respiratory Infections Group, ed. Cochrane Database Syst Rev 2017; 09. 10.1002/14651858.CD004417.pub5 PMC6372405 28881007

[37] Malani P, Solway E, Kirch M, Singer DC, Kullgren JT. Use and perceptions of antibiotics among US adults aged 50–80 years. Infect Control Hosp Epidemiol 2021; 42:628–9. 33541451 10.1017/ice.2021.19

[38] Moore A, McKelvie S, Glogowska M, Lasserson D, Hayward G. Infection in older adults: a qualitative study of patient experience. Br J Gen Pract 2020; 70:e312–21. 32253191 10.3399/bjgp20X709397 PMC7141814

[39] Webb TL, Sheeran P. Does changing behavioral intentions engender behavior change? A meta-analysis of the experimental evidence. Psychol Bull 2006; 132:249–68. 16536643 10.1037/0033-2909.132.2.249

[40] Liu T, Zhu Z, Thompson MP, et al Primary care practice telehealth use and low-value care services. JAMA Netw Open 2024; 7:e2445436. 39509127 10.1001/jamanetworkopen.2024.45436 PMC11544489

