# Supplementary material for: Comparing the Antimicrobial Resistance Crisis to the COVID-19 Pandemic: A Randomized Public Health Messaging Experiment
Source: Clin Infect Dis. 2026 Apr 8;83(1):e18–26. doi: 10.1093/cid/ciag110 (PMC13393112; doi:10.1093/cid/ciag110)
Supplement: ciag110_Supplementary_Data [file ciag110_supplementary_data.docx]

**Supplement**

**Title:** Comparing the antimicrobial resistance crisis to the COVID-19 pandemic: A randomized public health messaging experiment

**Authors:** Alistair Thorpe PhD^1^, Rachael A. Lee MD, MSPH^2,3^, Julia E. Szymczak PhD^4^, Madeline C. Farrell MSc, Isabelle Palmer BSc^5^, William B. Petty BSc^1^, Tyler Henderson^6^, Angela Fagerlin PhD^1,7^, Valerie M. Vaughn MD, MSc^8^

1. Department of Population Health Sciences, Spencer Fox Eccles School of Medicine at University of Utah, Salt Lake City, UT
2. Department of Medicine, Division of Infectious Diseases, UAB School of Medicine, AL
3. Department of Medicine, Division of Infectious Diseases, Birmingham VA Medical Center, Birmingham, AL
4. Department of Internal Medicine, Division of Epidemiology, Spencer Fox Eccles School of Medicine at University of Utah, Salt Lake City, UT
5. Spencer Fox Eccles School of Medicine at University of Utah, Salt Lake City, UT
6. Department of Psychology, Morehouse College, Atlanta, GA
7. Salt Lake City VA Informatics Decision-Enhancement and Analytic Sciences (IDEAS) Center for Innovation, Salt Lake City, UT
8. Department of Internal Medicine, Spencer Fox Eccles School of Medicine at University of Utah, Salt Lake City, UT

Corresponding author: Alistair Thorpe PhD; ([alistair.thorpe@hsc.utah.edu](mailto:alistair.thorpe@hsc.utah.edu))

**Supplement description**

This supplement contains full description of the survey items including the messages shown to each group for the present study and additional results tables, GROVE reporting checklist, and additional tables and figures.

**Survey**

**Study information:** We invite you to participate in a research study about decisions you make regarding your health. In this study, you will be asked some questions about your personal opinions, previous decisions, and experiences regarding antibiotics, common infections, and COVID-19. We will also ask some questions about yourself. If you agree to participate, we would like you to answer the questions on the following screens.

It will take approximately 15 to 20 minutes to complete this survey. You are free to skip any questions that you prefer not to answer. Compensation for participation will be provided in accordance with your panel agreement.

Every effort will be made to protect your privacy and confidentiality. We will not collect your name or any identifying information about you. Your participation will be completely anonymous and it will not be possible to link you to your responses. This study is not designed to benefit you directly. You have a choice about being in this study. You do not have to be in this study if you do not want to be.

The data we collect will be used for this study but may also be important for future research. Your data may be used for future research or distributed to other researchers for future study without additional consent if information that identifies you is removed from the data.

Taking part in this research study is completely voluntary. If you do not wish to participate in this study, simply click in the corner to close the web browser window.

You may have questions about your rights as someone in this study. If you have questions, you can call the University of Utah Institutional Review Board (the responsible Institutional Review Board) at 801-581-3655. Questions or concerns about this study or interest in the final results may be directed to Dr. Valerie Vaughn at valerie.vaughn@hsc.utah.edu. Thank you for taking part in this study.

What is your age?

Skip logic: if answer is <18 then skip to End of Survey.

How would you describe your gender identity?

- Female (1)
- Male (2)
- Transgender woman/Transwoman (3)
- Transgender man/Transman (4)
- Non-Binary/Third gender (5)
- Prefer not to say (6)
- Other please specify (7)

How would you describe your ethnic group or background? (Please select all that apply)

- American Indian or Alaskan Native (1)
- Asian or Asian American (2)
- Black or African American (3)
- Native Hawaiian or other Pacific Islander (4)
- White or European American (5)
- Other please specify (6)

Are you Hispanic or Latino/a or Latinx?

- No (1) | Yes (2)

What state do you live in?

↓ Alabama (1)…Wyoming (52)

There is a global health threat posed by bacteria that cannot be treated with antibiotics (i.e., antibiotic resistance). Please carefully consider the following information about antibiotics and antibiotic resistance. [*Respondents were then randomized to see one of the three messages below*]

- **Group 1 [Control]:** The impact of an antibiotic resistance crisis will be much more severe than anything we have seen before. It will also be much more difficult to control. We can all do our bit to prevent this crisis and the time for us to act is now. The most important thing we can all do is make sure we only use antibiotics when they are strictly necessary (when prescribed for a bacterial infection [e.g., pneumonia]). By doing so, we can prevent an antibiotic resistance crisis. With this in mind, please always remember that **most cold and flu symptoms are best treated at home by taking Tylenol or ibuprofen, and getting plenty of fluids and sleep.**
- **Group 2:** The COVID-19 pandemic has shown us how devastating a global health crisis can be. Every aspect of our daily lives had to change so that we could stay safe and protect the people we care about from COVID-19. We have all faced major challenges during this time and many of us have lost loved ones. This has given us first-hand experience of just how difficult these crises are to control once they have emerged.

The impact of an antibiotic resistance crisis will be much more severe than anything we have seen before—even COVID-19. It will also be much more difficult to control as it may not be possible to create new treatments against antibiotic resistant infections like we did with vaccines for COVID-19. However, we can all do our bit to prevent this crisis and the time for us to act is now. The most important thing we can all do is make sure we only use antibiotics when they are strictly necessary (when prescribed for a bacterial infection [e.g., pneumonia]). By doing so, we can prevent an antibiotic resistance crisis.

With this in mind, please always remember that **most cold and flu symptoms are best treated at home by taking Tylenol or ibuprofen, and getting plenty of fluids and sleep.**

-
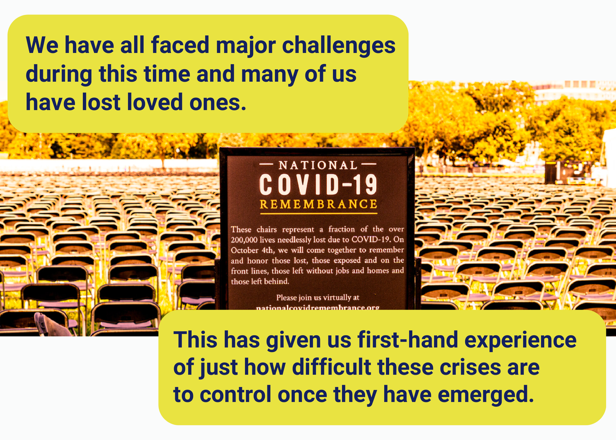

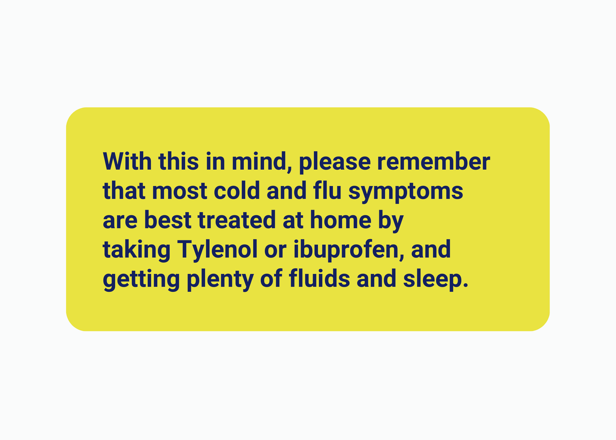

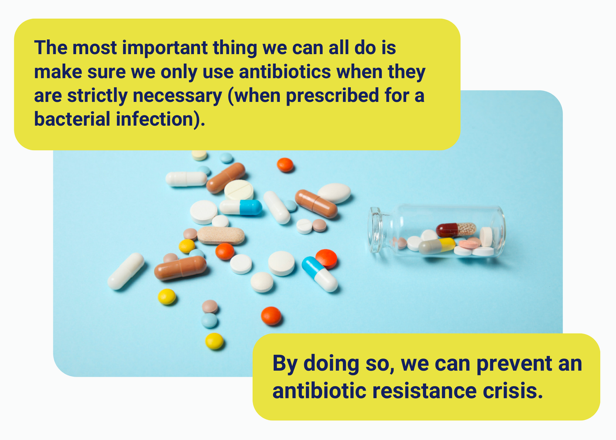

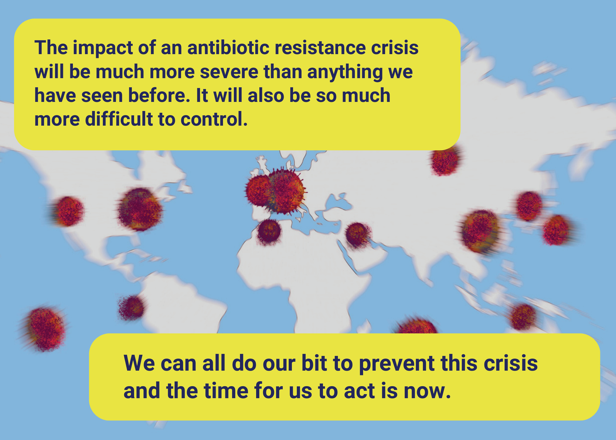

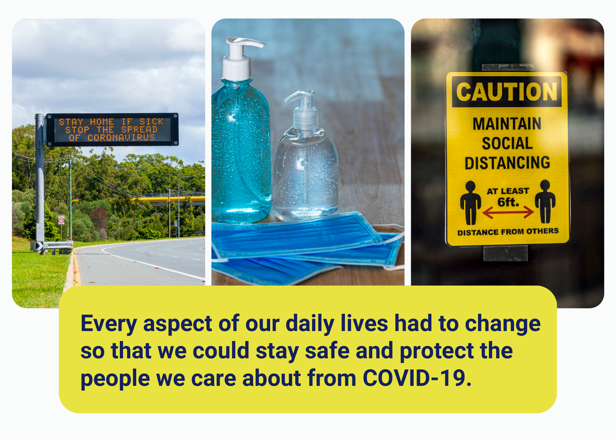

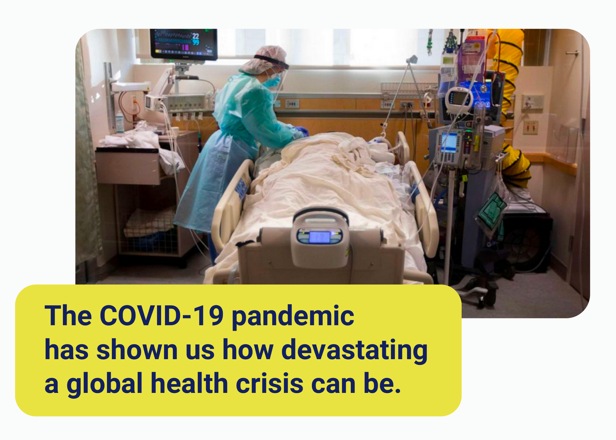
**Group 3:**

Please read the text below carefully and imagine that the situation described is real.

For over a week you have been feeling really ill and experiencing flu-like symptoms. You have had a couple of headaches and at one time had a high temperature (around 102°F). Repeated dry chesty coughing has caused you to develop a sore throat. Throughout the day your nose goes back and forth between being runny or blocked and you have been sneezing a lot. The symptoms persist during the day and throughout the evening. Your appetite is low, but you are still able to drink water and take small bites of food. Since the symptoms started, they have not really improved. You have

taken a test which shows that **you do not** have COVID-19.

At this point, do you think you would go to see a primary care clinician about these symptoms?

- Definitely WOULD NOT go to see a primary care clinician about these symptoms (1)
- Probably WOULD NOT go to see a primary care clinician about these symptoms (2)
- Probably WOULD go to see a primary care clinician about these symptoms (3)
- Definitely WOULD go to see a primary care clinician about these symptoms (4)

Would you want to get antibiotics for these symptoms?

- Definitely WOULD NOT want to get antibiotics (1)
- Probably WOULD NOT want to get antibiotics (2)
- Probably WOULD want to get antibiotics (3)
- Definitely WOULD want to get antibiotics (4)

Please read the text **about a different situation** below carefully and imagine that the situation described is real.

Yesterday you began to feel some discomfort in your side and lower back. After a few hours, this discomfort worsened and became very painful. You also have a high temperature (around 102°F) and feel the need to urinate often. When urinating, you experience a burning sensation and notice blood in your urine. You feel very weak, and you have no appetite for food. Today, as the symptoms have not improved, you have decided to see your primary care clinician. During the consultation, you discuss your symptoms, your previous illnesses and past medication usage. Your clinician takes your temperature and checks your blood pressure before asking you to provide a sample of urine. The urine test comes back positive for bacteria.

Your clinician tells you that the positive urine sample and your symptoms mean that you have a bacterial kidney infection. They explain that kidney infections, caused by bacteria travelling from your bladder into one or both of your kidneys, require prompt treatment with antibiotics for symptom relief and to prevent serious complications. Your clinician prescribes a 7-day course of antibiotics and some ibuprofen. They then mention that you should start to feel better soon after taking the

medication and the infection should have cleared up completely in about two weeks.

Would you take antibiotics if offered by your primary care clinician?

- Definitely WOULD NOT take antibiotics (1)
- Probably WOULD NOT take antibiotics (2)
- Probably WOULD take antibiotics (3)
- Definitely WOULD take antibiotics (4)

How concerned are you about experiencing short-term side effects from taking antibiotics?

- Not at all concerned (1)
- Slightly concerned (2)
- Somewhat concerned (3)
- Moderately concerned (4)
- Extremely concerned (5)
- Not sure (6)

How concerned are you about experiencing long-term side effects from taking antibiotics?

- Not at all concerned (1)
- Slightly concerned (2)
- Somewhat concerned (3)
- Moderately concerned (4)
- Extremely concerned (5)
- Not sure (6)

Now we would like to learn a little more about you. Thinking about your own personal history, experiences, and beliefs, please indicate your response to the following statements:

Sometimes, medical action is clearly necessary, and sometimes it is clearly NOT necessary. Other times, people differ in their beliefs about whether medical action is needed. In medical situations where it’s not clear, do you tend to lean towards taking action or do you lean towards waiting and seeing if action is needed? Importantly, there is no “right” way to be. Please answer on the 1-6 scale below:

- I lean toward waiting and seeing (1), (2), (3), (4), (5), I lean toward taking action (6)

Thinking about your own personal medical history, please indicate your response to the following statements:

In the past 12 months, I have taken antibiotics...

- 0 times (1), 1 time (2), 2 times (3), 3 times (4), 4 times (5), 5 or more times (6)

As far as you know, do you have any of the following health conditions at the present time?

|  | No, I do not have  this condition (0) | Yes, I have  this condition (1) |
| --- | --- | --- |
| Asthma, emphysema, or chronic bronchitis, COPD  *(other lung disease)* (1) | ° | ° |
| Arthritis or rheumatism (2) | ° | ° |
| Cancer, diagnosed in the past 3 years (3) | ° | ° |
| Diabetes (4) | ° | ° |
| Digestive problems  *(such as ulcer, colitis, or gallbladder disease)* (5) | ° | ° |
| Heart trouble  *(such as angina, congestive heart failure, or coronary artery disease, having a past heart attack)* (6) | ° | ° |
| HIV illness or AIDS (7) | ° | ° |
| Kidney disease (8) | ° | ° |
| Liver problems (such as cirrhosis) (9) | ° | ° |
| Stroke (10) | ° | ° |
| High blood pressure (hypertension) (11) | ° | ° |
| Very overweight or obese (12) | ° | ° |
|  | | |

Please indicate how much you agree or disagree with each statement. There are no right or wrong answers. Please answer in a way that reflects your own personal beliefs:

|  | Strongly disagree | Disagree | Somewhat disagree | Neither agree nor disagree | Somewhat agree | Agree | Strongly agree |
| --- | --- | --- | --- | --- | --- | --- | --- |
|  | (1) | (2) | (3) | (4) | (5) | (6) | (7) |
| People trust scientists a lot more than they should (1) | ° | ° | ° | ° | ° | ° | ° |
| People don’t realize just how flawed a lot of scientific research really is (2) | ° | ° | ° | ° | ° | ° | ° |
| A lot of scientific theories are dead wrong (3) | ° | ° | ° | ° | ° | ° | ° |
| Sometimes I think we put too much faith in science (4) | ° | ° | ° | ° | ° | ° | ° |
| Our society places too much emphasis on science (5) | ° | ° | ° | ° | ° | ° | ° |
| I am concerned by the amount of influence that scientists have in society (6) | ° | ° | ° | ° | ° | ° | ° |
|  | | | | | | | |

What is the highest level of schooling you have completed? (Check one)

- None (1)
- Elementary school (2)
- Some high school but no diploma (3)
- High school (Diploma or GED) (4)
- Some college, but no degree (5)
- Trade school (6)
- Bachelor’s degree (BS, BA, etc.) (7)
- Master’s degree (MA, MPH, etc.) (8)
- Doctoral/Professional degree (PhD, MD, etc.) (9)

How would you best describe the place where you live?

- Rural (1)
- Small city, e.g. less than 100,000 people (2)
- Suburban, near a large city (3)
- Mid-sized city, 100,000 to 1 million people (4)
- Large city, more than 1 million (5)
- Other, please specify (6)

Please take a moment to answer some questions about how you feel about reading health information. Please give the response that best matches how you feel.

How often do you have someone (like a family member, friend, hospital/clinic worker or caregiver) help you read instructions, pamphlets or other written health materials from your doctor or pharmacy?

- Never (1), Rarely (2), Sometimes (3), Often (4), Always (5)

How good are you at working with fractions?

- Not good at all (1), (2), (3), (4), (5), Extremely good (6)

How good are you at figuring out how much a shirt will cost if it is 25% off?

- Not good at all (1), (2), (3), (4), (5), Extremely good (6)

How often do you find numerical information to be useful?

- Never (1), (2), (3), (4), (5), Very often (6)

Which of the following describes your COVID-19 vaccination status?

- I have not received a COVID-19 vaccine (1)
- I have received at least one dose of either a Pfizer or Moderna vaccine (2)
- I have received at least one dose of either a Johnson and Johnson or Novavax vaccine (3)

I am proud that I am [not] vaccinated against COVID-19

- Do not agree at all (1), (2), (3), (4), (5), Very often (6)

How would you describe your political outlook with regard to social issues?

- Very liberal (1)
- Liberal (2)
- Slightly liberal (3)
- Moderate (4)
- Slightly conservative (5)
- Conservative (6)
- Very conservative (7)
- Prefer not to say (8)

How would you describe your political outlook with regard to social issues?

- Democrat (1)
- Republican (2)
- Independent (3)
- Liberal third party (4)
- Conservative third party (5)
- No political party affiliation (6)
- Prefer not to say (7)

**Debrief:** Thank you for your participation! You are now finished with this survey. In this study, we are interested in understanding how to communicate about the risks of antibiotic resistance and people’s attitudes and understanding of antibiotics. We greatly appreciate all your responses! We are learning a lot about these topics, and we hope to share our findings broadly soon.

For accurate, up-to-date information please see the following CDC websites:

- Antibiotic resistance: <https://www.cdc.gov/drugresistance/index.html>
- Patient resources about antibiotics: <https://www.cdc.gov/antibiotic-use/materials-references/index.html>
- COVID-19: <https://www.cdc.gov/coronavirus/2019-ncov/index.html>

Thank you for participating in this survey.

**GROVE Checklist**

**Checklist form - Guideline for RepOrting Vignette Experiments (GROVE)***

Note: this document was originally published in the journal ‘Patient Education and Counseling’ under Creative Commons Attribution 4.0 International License (see <http://creativecommons.org/licenses/by/4.0/>). No changes were made to the original document, which was included as Appendix 1 to the following article: Hillen, M.A., Visser, N.C., Labrie, N.H.M. et al. Development of GROVE: a Guideline for RepOrting Vignette Experiments conducted in a healthcare context. Pat Educ Couns (2025). DOI: <https://doi.org/10.1016/j.pec.2025.108750>

| **Criterion** | **Description** | **Location in manuscript where item is reported** | **Details on methodological approach**** |
| --- | --- | --- | --- |
| **1. Rationale** | Provide a rationale for the use of an experimental vignette-based design, including an explanation why the study could not be conducted in a non-simulated setting. | - **Discussion** | Our design allowed us to explore how whether framing an antibiotic resistance crisis in the context of the COVID-19 pandemic might affect intentions to seek care and take antibiotics for a viral upper respiratory tract infection whilst controlling for multiple factors (e.g., symptom type, symptom severity, type of clinical setting) that might also influence responses, that we could not achieve in a non-simulated setting. |
| **2. Vignette content** | Describe in detail how the vignette content was developed and refined, and explain any choices made. |  | *Report all relevant information below (sub-criteria 2.1-2.5)* |
| ***2.1. Clinical scenario*** | Describe and explain in detail how the healthcare scenario was developed and what it entailed. Include information about the sources used to inform vignette content, key characteristics of the portrayed characters, and the setting described in the vignette. | - **Procedure** - **Figure 1** - **Supplement** | The scenario was designed by the study team to represent a situation in which such a patient has a viral upper respiratory tract infection and should not receive antibiotics. After being randomized to one of three study-arms, respondents were shown a scenario describing themselves as experiencing flu-like symptoms for over a week with a negative COVID-19 test. |
| ***2.2. Manipulation &   standardization*** | Describe what the experimental manipulations are (i.e., operationalization of the phenomenon under study), detailing which elements of the scenario were varied and how. Also report how other elements in the vignette were kept constant and provide information on vignette duration or length. | - **Procedure** - **Figure 1** - **Supplement** | In a between-group experimental design, US adults were randomized to receive one of three messages: 1) written message describing the AMR crisis [control], 2) written message comparing AMR to the COVID-19 pandemic, 3) a series of poster-like graphics comparing AMR to the COVID-19 pandemic. Then all read the same hypothetical scenario describing a viral respiratory infection where antibiotics are not clinically indicated. |
| ***2.3. Mode of delivery*** | Describe and explain the delivery modality and provide any information necessary for replication. Explain choices regarding narrative perspective and amount of detail described. Describe how participants were introduced to the vignette and in which setting data were collected. | - **Methods** | The scenario was delivered to respondents in an online survey study and was viewed by respondents on their personal display system (e.g., monitor or laptop screen). |
| ***2.4. Expert   involvement*** | Explain who were involved in developing the vignettes, highlighting their particular expertise and contributions. | - **Procedure** | The study team developed the scenario with targeted input from clinicians, pharmacists, and social scientists |
| ***2.5. Pilot testing*** | Describe if, how, and when pilot testing was used in the vignette development process. Explain whether and how this affected the vignette content and format. | **N/A** | No pilot testing was conducted with the vignette |
| **3. Outcomes &   participant   instructions** | Explain the selected study outcome(s) for the vignette study, particularly how these outcome(s) relate to real-world outcomes of interest. | - **Measures** | The primary outcomes were whether they would a) want to visit a primary care clinician and b) desire to take antibiotics. These measures relate directly to patient preferences, desires, beliefs, and intentions which are known to influence their health behaviors. |
| **4. Vignette validity &   realism** | Report how manipulation success of the independent variable(s) of interest was evaluated (i.e., manipulation check). Also describe if and how realism and aspects of participant engagement with the scenario were assessed. | - **Results** | No specific manipulation checks were included. Respondent engagement with the scenario was assessed via responses to open-text questions. |
| **5. Participants** | Provide a rationale for the choice of study participants (e.g., analogue patients), both in relation to the target population and to the characters portrayed in the vignettes. | - **Introduction** | Our sample was chosen to reflect the US adult population. Thus the situation described in this vignette is clinically relevant to this population. |
| **6. Accessibility** | Include information on the availability of the final vignettes and pilot data for research, teaching, or commercial purposes. Detail any restrictions to access and (re)use of the vignettes and data. | - **Procedure** - **Figure 1** - **Supplement** | The complete final vignettes are provided in the supplementary materials. Access and reuse of the vignettes and associated data are permitted in accordance with the terms of the publishing license. |

*The order of reporting these criteria is intended to be flexible. Information can be combined or reorganized and information may be placed in any manuscript section, figure, table and or supplementary material, depending on the study content and journal requirements.

**Authors may report additional methodological details in this column beyond the information included in their main manuscript.

**Additional Tables and Figures**

| **Supplemental Table 1. Exploratory Multiple Regression Analyses of Factors Associated With Consulting Intentions (Model 1) and Desires for Antibiotics in the Viral Scenario** | | | | | | |
| --- | --- | --- | --- | --- | --- | --- |
|  | ***Model 1: Consulting intentions*** | | | ***Model 2: Desires for antibiotics*** | | |
|  | ***Estimates*** | ***CI*** | ***p value*** | ***Estimates*** | ***CI*** | ***p value*** |
| Age: 18-33 yrs | Reference level | | | | | |
| Age: 34-49 yrs | 0.05 | -0.11 to 0.21 | .542 | -0.05 | -0.21 to 0.11 | .561 |
| Age: 50- 64 yrs | -0.20 | -0.37 to -0.03 | **.021** | -0.26 | -0.43 to -0.09 | **.002** |
| Gender: Male | Reference level | | | | | |
| Gender: Female | -0.10 | -0.24 to 0.03 | .134 | -0.09 | -0.22 to 0.05 | .212 |
| Racial/Ethnic identity: Non-Hispanic White | Reference level | | | | | |
| Racial/Ethnic identity: Non-Hispanic Black | 0.10 | -0.10 to 0.30 | .328 | 0.18 | -0.02 to 0.37 | .078 |
| Racial/Ethnic identity: Hispanic | 0.14 | -0.05 to 0.32 | .150 | 0.12 | -0.06 to 0.31 | .197 |
| Racial/Ethnic identity: Any other identity | 0.17 | -0.09 to 0.43 | .196 | 0.15 | -0.10 to 0.41 | .244 |
| US Census Region: Northeast | Reference level | | | | | |
| US Census Region: Midwest | -0.01 | -0.23 to 0.21 | .951 | -0.11 | -0.33 to 0.10 | .305 |
| US Census Region: South | -0.04 | -0.22 to 0.14 | .674 | -0.12 | -0.30 to 0.06 | .181 |
| US Census Region: West | -0.15 | -0.37 to 0.06 | .158 | -0.22 | -0.43 to -0.01 | **.045** |
| Residence: Rural | Reference level | | | | | |
| Residence: Suburban | -0.00 | -0.17 to 0.16 | .982 | -0.02 | -0.19 to 0.14 | .801 |
| Residence: Urban | -0.09 | -0.27 to 0.08 | .299 | -0.03 | -0.21 to 0.14 | .707 |
| Educational attainment: High school or less | Reference level | | | | | |
| Educational attainment: Some college or trade | -0.17 | -0.34 to -0.00 | **.050** | -0.23 | -0.40 to -0.06 | **.008** |
| Educational attainment: ≥Bachelor’s | -0.15 | -0.32 to 0.02 | .093 | -0.21 | -0.38 to -0.04 | **.018** |
| Health literacy needs | 0.02 | -0.05 to 0.08 | .625 | 0.06 | -0.00 to 0.12 | .061 |
| Subjective numeracy | 0.07 | 0.01 to 0.13 | **.014** | 0.02 | -0.04 to 0.07 | .505 |
| Political stance on social issues | -0.05 | -0.10 to -0.00 | **.040** | -0.03 | -0.07 to 0.02 | .243 |
| Political Party: Democrat | Reference level | | | | | |
| Political Party: Republican | 0.01 | -0.20 to 0.23 | .894 | 0.12 | -0.09 to 0.33 | .282 |
| Political Party: Independent or 3rd Party | -0.17 | -0.34 to -0.00 | **.044** | -0.20 | -0.37 to -0.04 | **.016** |
| Political Party: Prefer not to say/None | -0.13 | -0.34 to 0.08 | .219 | -0.24 | -0.45 to -0.03 | **.023** |
| Antibiotic use over the past 12 months | 0.03 | -0.02 to 0.09 | .280 | 0.12 | 0.06 to 0.17 | **<.001** |
| Number of comorbid conditions | 0.02 | -0.01 to 0.06 | .249 | 0.03 | -0.01 to 0.06 | .142 |
| COVID-19 vaccination status: Unvaccinated (0 doses) | Reference level | | | | | |
| COVID-19 vaccination status: Vaccinated (≥1 dose) | 0.24 | 0.08 to 0.39 | **.003** | 0.16 | 0.00 to 0.31 | **.046** |
| Pride in vaccination status | 0.04 | 0.01 to 0.07 | **.004** | 0.03 | 0.00 to 0.06 | **.049** |
| Concern about Short-term ABX. side effects | 0.05 | -0.01 to 0.12 | .094 | 0.05 | -0.02 to 0.11 | .154 |
| Concern about long-term ABX. side effects | -0.01 | -0.07 to 0.05 | .809 | 0.01 | -0.05 to 0.07 | .775 |
| Medical maximizing | 0.13 | 0.09 to 0.17 | **<.001** | 0.12 | 0.07 to 0.16 | **<.001** |
| Disbelief in science | 0.03 | -0.02 to 0.08 | .296 | 0.02 | -0.04 to 0.07 | .516 |
| R^2^ | 0.154 | | | 0.193 | | |

| **Supplemental Figure 1. Primary outcome measures by group for age.** |
| --- |

Definitely

WOULD NOT

Probably

WOULD NOT

Probably

WOULD

Definitely

WOULD

AMR COVID-19 comparison

AMR COVID-19 comparison

AMR COVID-19 comparison

| **Supplemental Figure 2. Primary outcome measures by group for gender identity.** |
| --- |

AMR COVID-19 comparison

AMR COVID-19 comparison

AMR COVID-19 comparison

Definitely

WOULD NOT

Probably

WOULD NOT

Probably

WOULD

Definitely

WOULD

| **Supplemental Figure 3. Primary outcome measures by group for political party affiliation.** |
| --- |

AMR COVID-19 comparison

AMR COVID-19 comparison

AMR COVID-19 comparison

Definitely

WOULD NOT

Probably

WOULD NOT

Probably

WOULD

Definitely

WOULD

| **Supplemental Figure 4. Primary outcome measures by group controlling for COVID-19 vaccination status and pride.** |
| --- |

Definitely

WOULD

Probably

WOULD

Probably

WOULD NOT

Definitely

WOULD NOT

AMR COVID-19 comparison

AMR COVID-19 comparison

AMR COVID-19 comparison

AMR COVID-19 comparison

AMR COVID-19 comparison

AMR COVID-19 comparison
